# Exploring the effects of ecological activities during exposure to optical prisms in healthy individuals

**DOI:** 10.3389/fnhum.2013.00029

**Published:** 2013-02-12

**Authors:** Paola Fortis, Roberta Ronchi, Elena Calzolari, Marcello Gallucci, Giuseppe Vallar

**Affiliations:** ^1^Neuropsychological Laboratory, IRCCS Istituto Auxologico ItalianoMilano, Italy; ^2^Department of Psychology, University of Milano-BicoccaMilano, Italy

**Keywords:** prism adaptation, aftereffects, spatial neglect, right brain damage, rehabilitation, ecological, pointing

## Abstract

Prism adaptation improves a wide range of manifestations of left spatial neglect in right-brain-damaged patients. The typical paradigm consists in repeated pointing movements to visual targets, while patients wear prism goggles that displace the visual scene rightwards. Recently, we demonstrated the efficacy of a novel adaptation procedure, involving a variety of every-day visuo-motor activities. This “ecological” procedure proved to be as effective as the repetitive pointing adaptation task in ameliorating symptoms of spatial neglect, and was better tolerated by patients. However, the absence of adaptation and aftereffects measures for the ecological treatment did not allow for a full comparison of the two procedures. This is important in the light of recent findings showing that the magnitude of prism-induced aftereffects may predict recovery from spatial neglect. Here, we investigated prism-induced adaptation and aftereffects after ecological and pointing adaptation procedures. Forty-eight neurologically healthy participants (young and aged groups) were exposed to rightward shifting prisms while they performed the ecological or the pointing procedures, in separate days. Before and after prism exposure, participants performed proprioceptive, visual, and visual-proprioceptive tasks to assess prism-induced aftereffects. Participants adapted to the prisms during both procedures. Importantly, the ecological procedure induced greater aftereffects in the proprioceptive task (for both the young and the aged groups) and in the visual-proprioceptive task (young group). A similar trend was found for the visual task in both groups. Finally, participants rated the ecological procedure as more pleasant, less monotonous, and more sustainable than the pointing procedure. These results qualify ecological visuo-motor activities as an effective prism-adaptation procedure, suitable for the rehabilitation of spatial neglect.

## Introduction

Unilateral spatial neglect is a neuropsychological disorder that typically results from damage to the right cerebral hemisphere. Neglect is characterized by a failure to orient toward, respond to, and report stimuli that occur in the side of space contralateral to the side of the lesion (left, contralesional, in right-brain-damaged patients), and cannot be traced back to primary sensory-motor impairments. Patients with left neglect exhibit a large spectrum of symptoms involving different sensory modalities, internally generated images, and the contralesional side of the body. Spatial neglect may be qualified in terms of defective perceptual awareness, and impairment of the planning and execution of movements directed contralesionally (Bisiach and Vallar, 2000; Halligan et al., 2003; Husain, 2008; Heilman and Valenstein, 2011; Vallar and Bolognini, in press). In the past decades a number of rehabilitation procedures have been set up in order to ameliorate neglect symptoms (Parton et al., 2004; Luauté et al., 2006; Pizzamiglio et al., 2006; Arene and Hillis, 2007; Bowen and Lincoln, 2007; Adair and Barrett, 2008).

Adaptation to prisms displacing laterally the visual scene is a particularly promising technique: non-invasive, and easy to administer, it improves a wide range of neglect-related deficits (Rossetti et al., 1998, for a seminal study; see reviews in Redding and Wallace, 2006; Striemer and Danckert, 2010; Barrett et al., 2012). The standard procedure employed in prism interventions in neglect patients consists in the repetition of pointing movements toward visual targets. The same procedure has been typically used in healthy participants (Redding et al., 2005; Michel, 2006). Participants pointing to targets during prism exposure initially make a pointing error in the direction of the optical deviation (i.e., a rightward deviation for rightward shifting prisms, which are used for rehabilitating right-brain-damaged patients with left neglect). *Adaptation* to prisms is demonstrated by a progressive reduction of the pointing error throughout the exposure phase. Once prisms are removed, participants exhibit *aftereffects*, namely deviations in pointing and visual judgments (Redding and Wallace, 2006). Aftereffects have been mainly assessed through a *proprioceptive* test, in which blindfolded participants point to the subjective straight ahead, and a *visual-proprioceptive* test, in which they point toward visual targets, without viewing their arm. In these two tests participants make pointing errors in a direction opposite to that of the optical shift (i.e., leftwards for rightward deviating prisms). An additional measure of aftereffects is a *visual* test, in which participants verbally estimate the position of a visual target. Contrary to the shift induced in the pointing movements, the prism aftereffects observed in the visual test occur in the same direction of the optical displacement (i.e., rightward deviation for rightward shifting prisms, see Redding and Wallace, 2006, 2010).

Although repeated pointing movements have been the most widely used prism adaptation procedure for the rehabilitation of neglect patients, this method may be not optimal for long-term interventions, due to the repetitive and tedious nature of the pointings. The use of engaging and diverse visuo-motor tasks may be preferable for rehabilitation programs that require consecutive sessions for at least 2 weeks (Frassinetti et al., 2002; Fortis et al., 2010; Vangkilde and Habekost, 2010; Mizuno et al., 2011). A more varied procedure may provide a useful alternative if these can be shown to have similar beneficial effects.

In an early seminal study Stratton (1896, 1897) reported his own experience with prismatic lenses reversing upside down the visual scene; for 8 days he wore prismatic goggles during the day for several hours, while performing activities of daily life, such as walking indoor or outdoor (for reviews of early work see Day and Singer, 1967; Kornheiser, 1976). More recently, different tasks have been used in experiments performed in unimpaired participants and in patients with different types of brain-damage. These visuo-motor activities include movements for line bisection (Goedert et al., 2010; Fortis et al., 2011), locomotion/walking (Lackner, 1973; Morton and Bastian, 2004; Michel et al., 2008), and ball throwing (Martin et al., 1996; Fernández-Ruiz and Díaz, 1999). In a rehabilitation study, chronic neglect patients were exposed to prisms for 8 consecutive weeks, while tossing rings and performing a pegboard exercise; after prism adaptation the magnitude of leftward eye movements increased, and the center of gravity moved leftwards, indicating a reduction of left neglect (Shiraishi et al., 2008). In a recent study, we investigated whether a new *ecological* prism adaptation procedure could be effective in improving left neglect in a series of 10 right-brain-damaged patients (Fortis et al., 2010). The procedure consisted of a series of visuo-motor activities performed with daily life objects. In that study, patients underwent 20 sessions of prism adaptation during a period of 2 weeks, in which they performed the pointing task of Frassinetti et al. (2002) during 1 week and the ecological procedure during the other week, with the order of the two prism adaptation procedures being balanced across participants. Neglect signs improved after the first week and continued in the second week of treatment, with no differences between the two procedures (ecological vs. pointing). The main result is that the ecological prism adaptation procedure may provide a viable alternative to the traditional prism adaptation by repeated pointings. However, the study of Fortis et al. (2010) did not measure adaptation or aftereffects for the ecological task. Such measures are considered to be key indicators of the effectiveness of prism adaptation (Welch, 1978; Redding and Wallace, 1993). Thus, in the present study, we investigated whether the ecological procedure resulted in adaptation and aftereffects comparable to those previously demonstrated in the pointing task. Forty-eight healthy participants underwent 2 consecutive days of exposure to rightward shifting prism, performing the ecological task and the pointing task in separate days. The presence of aftereffects on each day was assessed by the proprioceptive, visual and visual-proprioceptive tests (Redding et al., 2005).

Both young and elderly participants entered the study. Age-dependent differences in sensorimotor adaptation have been reported, with elderly participants showing reduced rates of learning in visuomotor adaptation tasks (McNay and Willingham, 1998; Fernández-Ruiz et al., 2000; Bock, 2005; Bock and Girgenrath, 2006; Seidler, 2006), which are associated with a higher computational load (Bock and Schneider, 2002). Other studies show that sensorimotor adaptation is largely preserved in the elderly (Bock and Schneider, 2002; Roller et al., 2002). Particularly, in a sensorimotor (throwing) task, adaptation to laterally displacing visual prisms has been reported to be either preserved (Roller et al., 2002) or defective (Fernández-Ruiz et al., 2000). Conversely, aftereffects are preserved, or even larger, in elderly people (McNay and Willingham, 1998; Fernández-Ruiz et al., 2000; Roller et al., 2002; Bock, 2005). Experiments in healthy participants, using the paradigm of prism adaptation through repeated pointings, have been typically performed in young individuals (Berberovic and Mattingley, 2003; Michel et al., 2003, 2008; Loftus et al., 2009, 2008; Bultitude et al., 2012). In the present study the elderly group aimed at providing results suitable to be discussed with reference to the prism adaptation studies in the typically older brain-damaged patients. Finally, we administered a questionnaire at the end of each adaptation task, in order to assess the participants' level of satisfaction in performing the adaptation procedures, and the possible difficulties they had encountered in executing them.

## Materials and methods

Two groups of healthy participants (young and aged) were tested. The young group included 24 undergraduate students (12 females; age *M* = 24 years, *SD* = ±2.67, range 19–30; education *M* = 15 years, *SD* = ±1.37, range 13–17), enrolled in the Department of Psychology of the University of Milano–Bicocca, Italy. The aged group included 24 elderly participants (12 females; age *M* = 68 years, *SD* = ±5.74, range 57–79; education *M* = 13 years, *SD* = ±5.60, range 5–18), recruited from the inpatient population of the Neurorehabilitation Unit of the IRCCS Istituto Auxologico Italiano, Milan, Italy, with no history or evidence of neurological or psychiatric disorders. All participants had normal or corrected-to-normal vision, were right handed for writing, and were naïve to the purpose of the study. Handedness was assessed by the Edinburgh Handedness Inventory (Oldfield, 1971). The questionnaire included 10 items assessing hand preference, and two items assessing foot and eye preference, with scores 10 and 2 indicating complete right-handedness. The handedness scores were: *M* = 9.53 (*SD* = ±0.65, range 9–10) and *M* = 1.82 (*SD* = ±0.51, range 1–2) in the young group; *M* = 9.39 (*SD* = ±0.78, range 8–10) and *M* = 1.67 (*SD* = ±0.69, range 0–2), in the aged group. All participants gave informed consent prior to participating in the study. Students received course credits for their participation, which had been approved by the local Ethical Committees.

### Prism adaptation procedure

Participants underwent two prism adaptation sessions in 2 consecutive days, in which they completed a paradigm including: (1) a pre-exposure evaluation; (2) an exposure condition to base-left wedge prisms (Optique Peter, Lyon, France) displacing the visual field horizontally by 10° to the right; (3) a post-exposure evaluation, identical to the pre-exposure one.

During the exposure condition, participants performed the pointing adaptation task on 1 day and the ecological adaptation tasks on the other day. The order of the two prism adaptation procedures was counterbalanced: 24 participants (12 young and 12 aged) underwent the pointing adaptation task in the first day, and the ecological task in the following day; the other 24 participants (12 young and 12 aged) performed the adaptation tasks in the reverse order. Each adaptation task was carried out with the right arm.

### Pointing adaptation task

Participants sat at a table and positioned their right upper limb inside a 2-layer wooden box (32 cm high, 74 cm wide). The lower and upper surface of the box had a pentagonal shape with the base facing the participants' side. The pentagon's depth at the center (distance between the base and the vertex of the box) was 32 cm, and 19 cm at the lateral sides. Participants were asked to point with their right index finger to a target (the top of a red pen) presented by the examiner at the distal side of the box. They were instructed to perform one quick out-and-back movement. After each pointing, participants returned their hand to the starting position on the mid-line of the body, on the sternum, above the navel. A black cloth attached from the participant's neck to the upper surface of the box occluded the vision of the starting position of the arm. The pentagonal shape of the box occluded the view of the arm's movement up to the terminal part, so that only the right index finger emerging from the distal side of the box was visible. Ninety pointing movements were made. Target was presented in a pseudorandom fixed order 10° to the right or to the left of the participants' mid-sagittal plane of the trunk. The same number of trials was presented for each of the two target positions. The initial and last four pointing trials included two instances of the right and left target positions. The distal edge of the box was marked with angular gradations (degrees, °), attached on the upper side of the box on the examiner's side, which was not visible to participants. The distance between the target and the participants' finger was measured. A positive score denoted a rightward displacement with respect to the position of the target, a negative score a leftward displacement. The pointing adaptation task lasted 20 min, as in the study by Frassinetti et al. (2002), and was timed by stopwatch.

### Ecological adaptation task

During the ecological adaptation task participants performed 10 visuo-motor activities based on the manipulation of common daily life objects, selected from those employed by Fortis et al. (2010). The activities were presented in the following order: (1) collecting coins on the table and putting them in a money box, (2) selecting rings and bracelets from a box and wearing them on the left hand and fingers, (3) closing jars with the corresponding lids, (4) assembling jigsaw puzzles, (5) moving blocks from one compartment of a box to another compartment, as described in the Box and Block Test (Desrosiers et al., 1994), (6) sorting cards, (7) threading a necklace with 12 spools and rope, (8) copying a chessboard pattern on an empty chessboard, (9) serving a cup of tea, (10) composing a dictated word using letters printed on a square. Standardized instructions as to how to do each task were read to each participant before performing the experiment. During the ecological procedure the vision of the arm was available for the entire movement path. Immediately prior to and after the execution of the ecological activities, participants performed four pointing movements that were administered with an identical procedure as the one employed during the pointing adaptation task. The ecological adaptation task lasted 20 min, as the pointing task in the study by Frassinetti et al. (2002), and was timed by stopwatch.

### Pre- and post-exposure evaluation: aftereffect measures

Participants sat at a table with their head aligned with the mid-sagittal plane of their body, and stabilized by a chin-rest attached to the table. A transparent square panel (50 cm side) marked with a goniometry with lines radiating from −90° to +90° was placed on the table, centered on the participants' mid-sagittal plane. During the pre- and post-exposure evaluation, three aftereffects measures were assessed: proprioceptive, visual, and visual-proprioceptive. The three tasks were presented in counterbalanced order across participants. For the proprioceptive and the visual-proprioceptive tests participants were asked to perform fast and accurate pointing movements with their right upper limb. The participant's arm was positioned at the center of the panel, with the right hand resting on the starting location near their body and aligned with the mid-sagittal plane of the body. This served as a starting point for all movements.

#### Proprioceptive test

Participants were blindfolded and instructed to indicate the subjectively estimated position of their body midline on the panel surface. They performed 10 straight-ahead pointing movements. On each trial, the experimenter recorded the deviation of the finger position from the true objective body midline (°, degrees of visual angle).

#### Visual test

A red LED was mounted on a pulley (120 cm long, 1.5 cm wide) placed horizontally at the top of a black wooden box (35 cm high, 75 cm long, and 20 cm wide). The box was positioned in a darkened room at the distance of 85 cm from the participants' mid-sagittal plane. Two strings, placed on the two sides of the LED, were used to move it on the pulley. The speed of the LED movement was varied between trials in order to avoid counting strategies (Ronchi et al., 2011).

The visual test did not involve arm movements: participants were instructed to verbally stop the movement of the LED, when its position corresponded to their subjective mid-sagittal plane. The LED was moved 10 times: five times from right to left and five times in the opposite direction, starting with the right-to-left movement first, with respect to the participants' view. A centimeter attached to the pulley on the experimenter's side allowed for the recording of the deviation of the LED position from the center of the pulley corresponding to the participants' physical mid-sagittal plane (cm). Each measurement was then transformed in degrees of visual angle (°).

#### Visual-proprioceptive test

The same pulley-mounted LED box of the visual test was used. Participants performed 10 pointing movements on the panel surface to indicate the downward projected position of the LED. On each trial, the LED was placed in front of the participants' mid-sagittal plane, but participants were unaware of its position. The movement of the arm was occluded from vision by a 2-layer wooden box (30 cm high, 75 cm wide, and 50 cm deep) and by a black cloth attached from the participant's neck to the upper surface of the box. Participants were instructed to close their eyes between each trial to allow the experimenter to re-position the light.

### Questionnaire

A Likert-scale questionnaire was administered at the end of each day of the experiment, in order to assess the participants' experience of the adaptation tasks. Participants were required to indicate their level of agreement with each of 13 questionnaire statements. The scale ranged from 1 (“totally disagree”) to 7 (“totally agree”). The 13 items of the questionnaire (see Appendix) were then grouped into five general topics, referring to the pleasantness and feasibility (items 1–3), and monotony (4–5) of the task, to the motor discomfort caused by the activities (6–7), to prism-related discomfort (items 8–11), and to the willingness to repeat or extend the adaptation procedure over time (items 12–13).

### Statistical analysis

**Figure 1:**
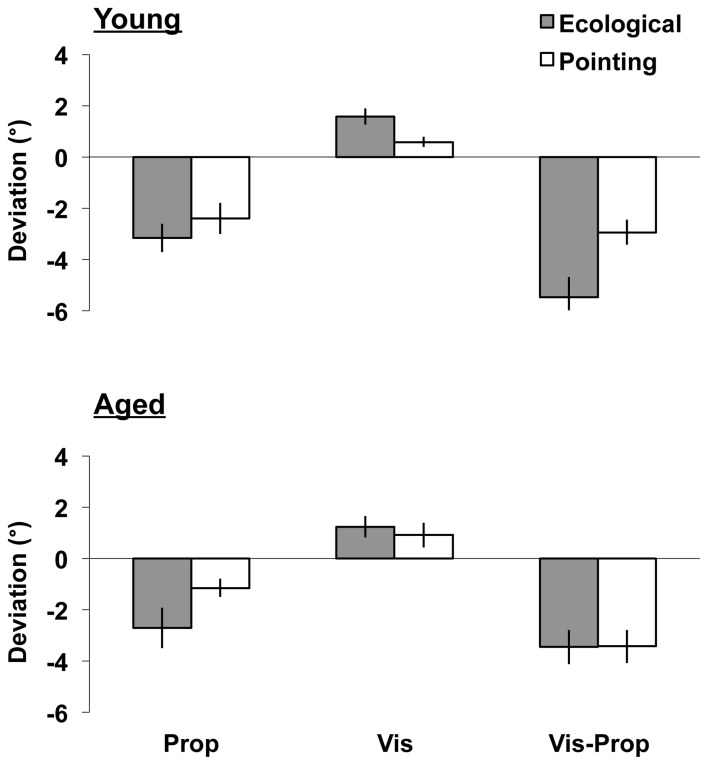
**Aftereffects.** Upper/lower panels: young/aged groups. Shifts (post-prism exposure *minus* pre-prism exposure mean pointing errors °, *SEM*; ±: rightward/leftward errors) induced by prism adaptation in the three aftereffects tests (proprioceptive, Prop: left bars; visual, Vis: middle bars; visual-proprioceptive, Vis-Prop: right bars), during the ecological (gray column) and the pointing (white column) adaptation procedures.

To evaluate to what extent participants adapted to prism exposure, by correcting the lateral deviation induced by the prismatic displacement (adaptation effect, see Redding et al., 2005; Redding and Wallace, 2006), the mean errors in the beginning (1–4) and end (87–90) four pointing trials of the prism exposure condition were computed during the pointing procedure. For the ecological task, the mean errors in the four pointing trials performed immediately before and after the visuo-motor adaptation activities were computed. A mixed-design analysis of variance (ANOVA) was performed with Time (Beginning/End four pointing trials) and Task (Ecological/Pointing) as the within-subjects factors, and Order of adaptation task (Pointing-Ecological/Ecological-Pointing) and Age (Young/Aged) as the between-subjects factors. Subsequent analyses were performed in order to quantify the presence and magnitude of aftereffects. The difference between the post- and the pre- exposure measures was computed, hereinafter referred to as *shift*. To compare the magnitude of aftereffects, an initial analysis was performed on the shifts induced in the proprioceptive, visual, and visual-proprioceptive tests. Since the prism aftereffects observed in the visual test occur in the direction opposite to those induced in the proprioceptive and visual-proprioceptive tests (Redding and Wallace, 2010), the sign of the shift of the visual test was inverted in the present analysis. A mixed-design ANOVA was performed on the shift, with Test (Proprioceptive, Visual and Visual-proprioceptive) and Task (Ecological/Pointing) as the within-subjects factors, and Order of adaptation task (Pointing-Ecological/Ecological-Pointing) and Age (Young/Aged) as the between-subjects factors. Secondly, to investigate the magnitude of the lateral shifts induced in the 2 days of prism exposure in the young and aged groups, three subsequent separate analyses, one for each test (Proprioceptive, Visual and Visual-proprioceptive), were performed on the shift, with Task (Ecological/Pointing) as the within-subjects factor, and Order of adaptation task (Pointing-Ecological/Ecological-Pointing), and Age (Young/Aged) as the between-subjects factors. In these analyses the visual shift was computed on the data, without sign inversion, as shown in Figure 1. Finally, the participants' mean responses for each topic of the questionnaire were analyzed by mixed-design ANOVAs with Task (Ecological/Pointing) as the within-subjects factor, and Order of adaptation task (Pointing-Ecological/Ecological-Pointing), and Age (Young/Aged) as the between-subjects factors. Significant differences were explored by Student-Newman–Keuls' *post-hoc* multiple comparisons.

## Results

### Adaptation as error correction effect

The main effect of Time [*F*_(1, 44)_ = 584.12, *p* < 0.001] was significant, showing that adaptation occurred so that the prism-induced rightward deviation in the initial four trials (*M* = 3.54°, *SD* = ±1.15) of prism exposure was corrected in the last four trials (*M* = 0.12°, *SD* = ±0.53). The main effect of Task [*F*_(1, 44)_= 4.72, *p* = 0.035] was also significant, indicating overall more deviation in the pointing task (*M* = 1.95°, *SD* = ±0.77) than in the ecological task (*M* = 1.71°, *SD* = ±0.91). Importantly, the interaction between Time and Task was not significant [*F*_(1, 44)_ = 0.07, *p* = 0.79], indicating that the ecological (initial trials *M* = 3.34° *SD* = ±1.40; last trials *M* = −0.02°, *SD* = ±0.89) and the pointing (initial trials: *M* = 3.65°, *SD* = ±1.53; last trials: *M* = 0.26°, *SD* = ±0.52) tasks induced the same magnitude of adaptation effect. Furthermore, this interaction did not depend on Age [Time by Task by Age: *F*_(1, 44)_ = 0.44, *p* = 0.509], indicating that the ecological and the pointing tasks were equally effective in the young and in the aged groups. No interaction was found between Time and Age [*F*_(1, 44)_ = 0.60, *p* = 0.445], indicating equally strong adaptation in the young and aged groups, when averaging across tasks. The Task by Order of adaptation task interaction [*F*_(1, 44)_ = 46.79, *p* < 0.001], and the Task by Time by Order of adaptation task interaction [*F*_(1, 44)_ = 7.34, *p* = 0.010] were significant. Because the two tasks (ecological, pointing) were performed in different days, with the order specified in the Order of adaptation task factor, the interaction between Task and Order of adaptation task effectively reflected differences in the overall deviation between the 2 days in which adaptation was measured. The deviation on the beginning and the end trials (adaptation effect) was greater in the first day than in the second day. Inspection of the means revealed that this effect was driven by less rightward mean deviation in the beginning pointing errors of the second day (*M* = 3.00°, *SD* = ±1.34) compared to the first day (*M* = 4.09°, *SD* = ±1.39, *p* < 0.001). Similarly, the last mean pointing errors of the second day (*M* = −0.11°, *SD* = ±0.68) were less rightward deviated than the last mean pointing errors of the first day of prism exposure (*M* = 0.34°, *SD* = ±0.74, *p* < 0.001). The Age by Order of adaptation task interaction [*F*_(1, 44)_ = 5.25, *p* = 0.027] was also significant. *Post-hoc* comparisons revealed a trend toward significance for a greater overall mean deviation in the old group, who performed the task in the order ecological-pointing (*M* = 2.31°, *SD* = ±0.75), than in the order pointing-ecological (*M* = 1.55°, *SD* = ±0.22, *p* = 0.073). A similar trend of a greater overall deviation in the old group, who performed the task in the order ecological-pointing (*M* = 2.31°, *SD* = ±0.75), compared to the young group with the same order (*M* = 1.61°, *SD* = ±0.75), was found. No other significant main effects or interactions were found in the analysis (*p* > 0.054, for all tests).

### Pre-post test differences: aftereffects measures

The initial analysis compared the shift (the difference between the post- and the pre- exposure measures) induced in the proprioceptive, visual, and visual-proprioceptive tests following the ecological and the pointing adaptation tasks in the young and aged participants (see Figure 1). The main effect of Test [*F*_(2, 88)_ = 21.63, *p* < 0.001] was significant. *Post-hoc* comparisons showed that prism exposure induced a greater lateral deviation in the visual-proprioceptive test, followed by the proprioceptive, and the visual tests (*p* < 0.003, for all tests). Importantly, the main effect of Task was significant [*F*_(1, 44)_ = 8.75, *p* = 0.005] revealing that the magnitude of aftereffects varied according to the task performed during the adaptation phase. Inspection of the means showed a greater deviation after the ecological than the pointing adaptation task (see Figure 1). Furthermore, the Task by Test by Age interaction was significant [*F*_(2, 88)_ = 3.26, *p* = 0.043], indicating that the ecological and the pointing tasks differently affected the aftereffects in the young and aged groups, as further assessed in the following three ANOVAs, one for each test. No other significant main effects or interactions were found in the analysis (*p* > 0.124, for all tests).

### Proprioceptive test

The shift after prism exposure was significant (comparison of mean shift against zero; i.e., intercept of the ANOVA, [*F*_(1, 46)_ = 50.29, *p* < 0.001]), showing that exposure to rightward shifting prisms induced a significant leftward deviation in the proprioceptive measures. The main effect of Task was significant [*F*_(1, 44)_ = 4.85, *p* = 0.033], revealing that the magnitude of the aftereffects varied according to the task performed during the adaptation phase. As shown in Figure 1 (left bars), the ecological adaptation task brought about a greater leftward deviation than the pointing task in both the young and the aged groups. No other significant main effects or interactions were found in the analysis (*p* > 0.209, for all tests).

### Visual test

**Figure 2:**
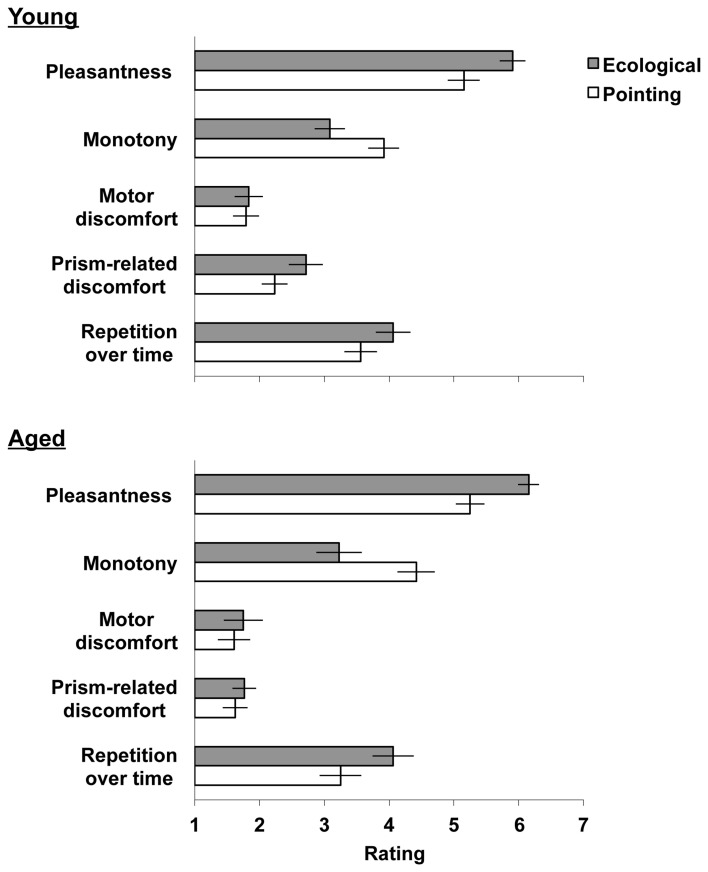
**Mean level of agreement scores (*SEM*) of the ecological (gray bars) and the pointing (white bars) prism adaptation procedures by the five questionnaire topics.** Scale range: 1 (“totally disagree”) −7 (“totally agree”).

The shift after prism exposure was significant (comparison of mean shift against zero; i.e., intercept of the ANOVA [*F*_(1, 44)_ = 30.82, *p* < 0.001]), showing that exposure to rightward shifting prisms induced a significant rightward deviation in the visual measures. The main effect of Task showed a trend toward significance [*F*_(1, 44)_ = 3.79, *p* = 0.058] revealing that the magnitude of the aftereffects varied according to the task performed during the adaptation phase. As can be seen in Figure 2 (central bars), there was a trend toward a greater rightward deviation after the ecological adaptation task than after the pointing adaptation task in both the young and the aged groups. The Age by Order of adaptation interaction [*F*_(1, 44)_ = 3.90, *p* = 0.055] showed a trend toward significance. Inspection of the means revealed a greater mean deviation in the old group who performed the task in the order pointing-ecological (*M* = 1.72°, *SD* = ±1.35) than in the order ecological-pointing (*M* = 0.43°, *SD* = ±1.35). No other main effects or interactions were significant (all *p* > 0.173).

### Visual-proprioceptive test

The shift after prism exposure was significant (comparison of mean shift against zero; i.e., the intercept of the ANOVA [*F*_(1, 44)_ = 124.26, *p* < 0.001]), showing that exposure to rightward shifting prisms induced a significant leftward deviation in the visual-proprioceptive measures. The main effect of Task was significant [*F*_(1, 44)_ = 4.17, *p* = 0.047], and the interaction of Task by Age was close to significance [*F*_(1, 44)_ = 4.01, *p* = 0.051]. As shown in Figure 1 (right bars), inspection of the means revealed that the ecological task brought about a greater leftward deviation in the young group (ecological: *M* = −5.48°, *SD* = ±3.88; pointing: *M* = −2.93°, *SD* = ±2.39), whereas a much smaller difference between the two tasks was found in the group of aged participants (ecological: *M* = −3.45°, *SD* = ±3.28; pointing: *M* = −3.43°, *SD* = ±3.18). In addition, the ecological task brought about a greater shift in the young group than in the elderly group (young: *M* = −5.48°, *SD* = ±3.88; elderly: *M* = −3.45°, *SD* = ±3.28). No other significant main effects or interactions were found in the analysis (*p* > 0.140, for all tests).

### Questionnaire

Figure 2 shows that both the young and the elderly groups of participants preferred performing the ecological adaptation task, as they found it more pleasant, less monotonous and more desirable to repeat for prolonged periods. Adaptation to prisms was well tolerated by both groups, with a slightly increased prism-related discomfort after the ecological procedure for the young group only.

For the pleasantness of the task, the main effect of Task was significant [*F*_(1, 44)_ = 33.26 *p* < 0.001], showing that the ecological task was considered more pleasant than the pointing adaptation task. No other significant main effects or interactions were found in the analysis (*p* > 0.314, for all tests).

As for the monotony of the task, the main effect of Task was significant [*F*_(1, 44)_ = 19.95, *p* < 0.001]. The Task by Order of adaptation task [*F*_(1, 44)_ = 4.68, *p* = 0.036] was also significant. *Post-hoc* comparisons revealed that the ecological task performed by the pointing-ecological group in the second day (level of agreement *M* = 2.63, *SD* = ±1.34) was considered less monotonous than the pointing task performed in the first day (*M* = 4.13, *SD* = ±1.31); similarly it was considered less monotonous than the ecological task (*M* = 3.69, *SD* = ±1.34) and the pointing task (*M* = 4.20, *SD* = ±1.31) performed by the ecological-pointing group (*p* < 0.01, for all tests). Thus, when the ecological task was performed after the pointing task it was considered less monotonous. No other significant main effects or interactions were found in the analysis (*p* > 0.071, for all tests).

As for the discomfort related to the motor activities, no significant main effects or interactions were found in the analysis (*p* > 0.494, for all tests) suggesting that young and elderly participants experienced pain in the arm or in the body neither after the ecological nor after the pointing adaptation task.

As for the prism-related discomfort, the main effects of Task [*F*_(1, 44)_ = 16.07, *p* < 0.001] and of Age [*F*_(1, 44)_ = 7.00, *p* = 0.012] were significant, and the interaction of Task by Age showed a trend toward significance [*F*_(1, 44)_ = 3.68, *p* = 0.062]. Inspection of the means revealed that young participants experienced greater side effects of prisms after the ecological adaptation task (*M* = 2.91, *SD* = ±1.31) than after the pointing adaptation task (*M* = 2.38, *SD* = ±1.01). This difference was smaller in the aged group of participants (ecological task *M* = 1.92, *SD* = ±1.08; pointing task *M* = 1.73, *SD* = ±0.97). Nevertheless, responses remained at the disagreement level, suggesting that the execution of both adaptation procedures was overall well tolerated by either group of participants. No other significant main effects or interactions were found in the analysis (*p* > 0.454, for all tests).

Lastly, for the items that assessed the willingness to extend the adaptation procedure over time, the main effect of Task [*F*_(1, 44)_ = 10.14, *p* < 0.001] was significant, showing that participants preferred to perform the ecological task for a longer period of time. No other significant main effects or interactions were found in the analysis (*p* > 0.157, for all tests).

## Discussion

In the present study we assessed whether a new ecological procedure, performed during exposure to rightward shifting prisms, could generate adaptation and aftereffects, in two groups of young and elderly healthy participants. To this end, we compared the effects induced by the ecological procedure with those induced by the pointing task, a standard procedure employed in prism adaptation studies (Redding et al., 2005; Redding and Wallace, 2010).

### Adaptation effect

Performing ecological or pointing adaptation tasks induces comparable corrections of the pointing movements during prism exposure, resulting in spatially accurate performance at the end of the exposure phase (adaptation effect), with no age differences. Indeed, in the beginning trials of the exposure condition, participants make pointing errors that are rightward deviated from target location as a consequence of the optical displacement. Errors are similarly reduced at the end of the exposure phase following either adaptation tasks. These results are in line with the evidence that elderly healthy participants exhibit adaptation effects (achieved through a throwing task) to prisms displacing the visual scene laterally, comparable to those of young participants (Roller et al., 2002). In another study (Fernández-Ruiz et al., 2000), using a similar paradigm, the aged group adapted more slowly than the young group, but both achieved the same adaptation levels. The present results extend to the ecological and pointing tasks that there are no-age-related differences in healthy participants as for adaptation effects.

### Aftereffects measures

The ecological and the pointing procedures bring about significant deviations in the three aftereffects measures in both the young and the aged groups of participants. Specifically, the visually-guided movements performed by participants during the ecological tasks cause deviations in the three aftereffects measures in the same direction as those previously reported after exposure to rightward shifting prisms, with adaptation having been achieved through repeated pointings (Redding et al., 2005). Strikingly, we found greater aftereffects following the ecological task: particularly, in the proprioceptive task in both the young and the aged groups of participants, and in the visual-proprioceptive task in the young group. For the visual task a similar trend was found in both age groups.

The increased magnitude of the three aftereffects following the ecological procedure is of interest, since it provides some hints as to the factors modulating the building up of aftereffects. Several differences between the ecological and the pointing tasks may underlie this result.

The pointing task is based on timed and interrupted movements; it requires to point and return to the rest position and to wait for a signal by the experimenter, to execute the next trial. Conversely, during the ecological task, participants perform free and more varied patterns of movements, in which they manipulate several everyday objects. This more varied manipulation may have required the allocation of attentional resources more than in the pointing task. There is evidence that a task such as mental arithmetic during adaptation brings about a reduction of visual aftereffects, putatively due to the allocation of attentional resources to the secondary task (Redding et al., 1985). In the present study, the more varied ecological task may have required the allocation of more attentional resources than the repetitive pointing task, resulting in enhanced aftereffects.

Additionally, participants may have been more engaged and motivated during the ecological than during the pointing procedure. The results of the questionnaire are by and large in line with these conclusions. The role of all these factors was not addressed in the present study, which aimed at assessing the aftereffects brought about by the two prism adaptation activities. These issues may be investigated in future specific studies.

Some differences in the magnitude of the aftereffects in the young and in the aged groups of participants were also found. The visual-proprioceptive shift in the ecological task was greater in the young than in the aged group. The available literature provides conflicting evidence. One prism adaptation study found larger aftereffects in the elderly group (Fernández-Ruiz et al., 2000 throwing a ball, and testing a visuo-proprioceptive shift), while another, using the same prism adaptation method, found no age-related differences (Roller et al., 2002). Overall, our results in the pointing task agree by and large with the conclusion that aftereffects are comparable in young and elderly participants (see Roller et al., 2002, who used the task of ball throwing, broadly similar to the present pointing task). The greater visuo-proprioceptive aftereffects exhibited by young participants after ecological adaptation might tentatively indicate a more effective visuo-motor integration in the young group, possibly supported by relatively more efficient cognitive abilities (Redding et al., 1985; Bock and Schneider, 2002), involved in the more varied ecological procedure, that is open to strategic effects (e.g., choosing how to perform the task).

Another factor that may modulate age-related differences in prism adaptation involves pre-existing biases of spatial attentional systems. Young healthy participants show a leftward bias in bisection tasks (*pseudoneglect*), which diminishes in aged participants, with a relative rightward deviation (Jewell and McCourt, 2000, for review; Schmitz and Peigneux, 2011), although there is also evidence for a stability of left pseudoneglect in the life span (see Beste et al., 2006, for visual line bisection; Brooks et al., 2011, for tactile rod bisection). This age-related difference may reflect a minor hemispheric asymmetry of spatial functions in elderly participants (Cabeza, 2002; Dolcos et al., 2002), which results in a reduction of the leftward deviation. Goedert et al. (2010), using a line bisection task, found rightward and leftward aftereffects in elderly participants, after exposure to leftward and rightward deviating prisms respectively, and no left pseudoneglect. Conversely, young participants, who showed left pseudoneglect, exhibited (rightward) aftereffects only after exposure to leftward deviating prisms, although a trend with rightward deviating prisms was found. In the present study, only rightward deviating prisms were used, and we found aftereffects in both age groups, in line with previous evidence (Fernández-Ruiz et al., 2000; Roller et al., 2002). It should be noted, however, that the tasks were different [line bisection (Goedert et al., 2010) vs. pointing and ecological activities in the present study, more similar in this respect to those of Roller et al. (2002), and of Fernández-Ruiz et al. (2000)], preventing a direct comparison.

### Implication for studies in patients with left neglect

The finding of consistent aftereffects following the ecological procedure has potentially relevant implications for the rehabilitation of neglect patients. The suggestion has been made that the recovery of spatial neglect after a prism adaptation treatment is related to the magnitude and the duration of the aftereffects. In a group study (Fortis et al., 2010) of 10 right-brain-damaged patients with left neglect, who underwent 10 sessions of prism adaptation performed with a pointing task over a period of 1 week, the size and the duration of the visual-proprioceptive aftereffects were related to the improvement of neglect, as assessed by cancellation tasks; the persistence and magnitude of the long-term aftereffects even mediated the improvement of functional abilities of neglect patients, as assessed by the Functional Independence Measure (FIM™) scale (Tesio et al., 2002). In a single session study performed in 13 right-brain-damaged patients, those participants who showed prism adaptation-induced improvement in target cancellation exhibited larger proprioceptive aftereffects than those patients whose cancellation performance did not improve; conversely, the visual-proprioceptive aftereffects were minor in size, and unrelated to recovery from neglect (Sarri et al., 2008). Other reports appear to relate the improvement of neglect after prism exposure to the adaptation effect (i.e., error correction during the exposure phase), rather than to the aftereffects. In two studies (Frassinetti et al., 2002; Serino et al., 2007) patients who show no or little adaptation effects exhibit less improvement of the neglect deficit; in one study (Serino et al., 2006) the improvement of neglect is related to the development of prism adaptation during 1 week of treatment, rather than to the magnitude of aftereffects. In functional models of prism adaptation (Redding and Wallace, 2006), the improvement of left spatial neglect is related to the aftereffects (leftward visuo-proprioceptive, and proprioceptive; rightward visual) induced by exposure and adaptation to rightward displacing visual prisms. The rightward “visual shift would bring the neglected left-hemispace into the narrowed task-work space, thereby ameliorating neglect,” and the “leftward shift in origin of proprioceptive reference frame would produce more responses in the neglected hemispace” (*loc. cit*., pp. 14–15). The present findings of greater aftereffects following the ecological tasks raise the possibility that the ecological procedure for prism adaptation may even improve the rehabilitation outcome of neglect patients, as compared with prism adaptation through pointings (Frassinetti et al., 2002). Future studies should test whether the present findings in healthy participants generalize to neglect patients.

Importantly, there are differences in the magnitude of the aftereffects found in right-brain-damaged patients with left spatial neglect and in healthy participants. After adaptation to rightward displacing prisms through repeated pointings patients with left neglect show disproportionately large leftward aftereffects (as assessed by the proprioceptive straight ahead task), and appear unaware of the optical effects of prisms (Michel et al., 2007, for related evidence in healthy participants; Rossetti et al., 1998; Rode et al., 2003). The possibility may be considered that the larger leftward aftereffects (i.e., the reduction of a disproportionate rightward proprioceptive shift) found in right-brain-damaged patients with left neglect represent a reduction of a manifestation of neglect itself, namely a rightward bias in the subjective straight ahead, as assessed by the proprioceptive task (Heilman et al., 1983). In line with this view, Sarri et al. (2008) found in right-brain-damaged patients with left spatial neglect, as compared with neurologically unimpaired control participants, disproportionate leftward aftereffects of prism adaptation on the disproportionately rightward deviated proprioceptive straight ahead, but not on a task requiring pointing to visual targets located on the mid-sagittal plane. These findings comport with the view that a basic deficit of neglect is an ipsilesional deviation of the egocentric reference frame, originally proposed by Ventre et al. (1984), and subsequently revived by Karnath (1994, with a rightward visual shift). Other studies in right-brain-damaged patients with left neglect, however, have questioned these findings and interpretations, showing that the subjective straight ahead is largely preserved (Farnè et al., 1998), and its shifts (found to occur both rightwards and leftwards) unrelated to the main clinical manifestations of left spatial neglect, such as defective target cancellation or drawing, and line bisection performance (Chokron and Bartolomeo, 1997; Hasselbach and Butter, 1997; Perenin, 1997; Bartolomeo and Chokron, 1999). Furthermore, patients with parietal damage and optic ataxia without unilateral spatial neglect show an ipsilesional deviation of the egocentric reference (Perenin, 1997). In sum, while right-brain-damaged patients with left neglect show disproportionate leftward aftereffects in the proprioceptive task after prism adaptation, it is dubious that this shift is a cardinal manifestation of spatial neglect. Future studies in brain-damaged patients may explore the magnitude of aftereffects after pointing and ecological adaptation procedures.

Results from the questionnaire show that the ecological procedure is considered more pleasant and interesting to perform than the pointing task. Participants evaluate the ecological visuo-motor activities less repetitive, more enjoyable, and easier to perform. They are also more willing to repeat them over time. Increasing the patients' compliance to the therapy may allow a higher number of brain-damaged patients to go through the whole training, as a result of a greater and active participation in the activities aimed at inducing adaptation and aftereffects. Previous studies have indeed shown that, in general, the patients' compliance with the treatment can improve the rehabilitation outcome, including measures of functional independence, and can even result in a shorter hospitalization time (Maclean and Pound, 2000; Lenze et al., 2004).

A number of studies have shown that multiple sessions are effective for rehabilitating spatial neglect. In the study by Fortis et al. (2010) 2 weeks of treatment were more effective than 1 week, which nevertheless brought about some improvement. A treatment of at least 2-weeks (10 sessions) appears to be an effective standard (Frassinetti et al., 2002; Humphreys et al., 2006, one patient, 5 weeks of treatment, with two sessions weekly; Serino et al., 2006, 2007; Shiraishi et al., 2008, 8 weeks of treatment, with about four sessions weekly; Vangkilde and Habekost, 2010; Làdavas et al., 2011; Nijboer et al., 2011, one patient, 3 months with daily sessions). Rehabilitation studies reporting negative findings in neglect patients employed treatments with shorter duration (Nys et al., 2008, 4 days), or weaker displacing lenses (Turton et al., 2010, 6° lenses). Importantly, long-term training has shown positive impact on functional abilities of everyday life, as assessed by Activities of Daily Living (ADL) Scales: the FIM™ (Tesio et al., 2002) scale (Fortis et al., 2010; Mizuno et al., 2011); the Barthel Index (Mahoney and Barthel, 1965), and Lawton's IADL scale (Shiraishi et al., 2008, 2010). In sum, it is preferable to use an adaptation procedure more appreciated by patients, given the length (at least 2 weeks) of the treatment.

Finally, the ecological adaptation procedure opens up new possibilities for extending the prism adaptation-based rehabilitation of neglect patients for longer time periods. Indeed, ecological visuo-motor activities may be easily designed for home-based rehabilitation programs, customized to the domestic environment. This appears to be an especially important development, considering that it may allow for long-term programs that are not feasible in inpatient rehabilitation facilities, due to the increasing trends (Taylor et al., 2010) toward shorter hospitalization periods.

### Conflict of interest statement

The authors declare that the research was conducted in the absence of any commercial or financial relationships that could be construed as a potential conflict of interest.

## Appendix

Questionnaires performed after the ecological procedure (version A) and after the pointing procedure (version B).

**A:** How did you experience wearing the goggles while you were manipulating the objects?
***B:** How did you experience wearing the goggles while you were pointing to the pen?*
  1. It was enjoyable
  2. It was interesting
  3. It was easy to perform
  4. It was boring
  5. It was repetitive
  6. It was painful for my arm
  7. It was tiring to maintain the posture
  8. My eyes were getting tired
  9. It made me dizzy
  10. It made me sick
  11. I visually perceived objects distorted
  12. I would have liked to continue the activity
  13. I would like to participate in future experiments with the same procedure
